# Editorial on the Special Issue “Fluorescence Imaging and Analysis of Cellular Systems”

**DOI:** 10.3390/jimaging10110293

**Published:** 2024-11-18

**Authors:** Ashutosh Sharma

**Affiliations:** Department of Chemistry, University of Illinois Chicago, Chicago, IL 60607, USA; sharma61@uic.edu

Fluorescence imaging has indeed become a cornerstone in modern cell biology due to its ability to offer highly sensitive, specific, and real-time visualization of cellular structures and dynamic processes [1]. This technique leverages the ability of fluorescent molecules (fluorophores/quantum dots) to absorb light at one wavelength and emit it at a longer wavelength, allowing researchers to label and track individual proteins, organelles, or even entire cellular pathways [2,3]. By tagging specific cellular components with fluorescent dyes or genetically encoding fluorescent proteins like G/RFP (Green/Red Fluorescent Protein), fluorescence imaging enables the real-time observation of intracellular events such as protein interactions, signaling pathways, and organelle dynamics [4,5]. Advances in fluorescence microscopy, including confocal and super-resolution methods, have further enhanced the spatial and temporal resolution of cellular imaging, making it a powerful approach for studying complex cellular systems [6,7]. Fluorescence-based techniques are integral to understanding various biological phenomena, from basic cell function to disease pathogenesis, enabling the high-resolution analysis of processes like gene expression, molecular trafficking, and cell division [8].

The study on “UpU-Net approaches for background emission removal in fluorescence microscopy” introduces a novel application of a U-Net-like architecture to effectively remove background emission from microscopy images. This method leverages Perlin noise as a simulated model for background emission, which closely mimics the actual noise patterns observed in fluorescence microscopy. By integrating this simulated noise, the deep learning model successfully addresses both background emission and additional noise perturbations, such as Gaussian and Poisson noise, significantly enhancing image clarity. The proposed UpU-Net architecture outperforms the traditional U-Net, offering improvements in both performance and computational efficiency (Contribution 1). One key achievement of this model is its ability to improve particle recognition in microscopy images, achieving a 90% accuracy rate in detecting actual particles. This advancement is crucial for precise image analysis in biological research. Additionally, the study suggests that the success of using Perlin noise as a background model opens opportunities for more sophisticated 3D data generation and broader applications in microscopy imaging. The research sets the stage for future innovations in noise reduction techniques and their application to more complex imaging tasks. 

The article titled “Gadolinium and Bio-Metal Association: A Concentration Dependency Tested in a Renal Allograft and Investigated by Micro-Synchrotron XRF” examines the distribution and concentration of Gadolinium (Gd) in a transplanted renal allograft (Contribution 2). The study reveals a heterogeneous distribution of Gd, predominantly in fibrotic areas and regions with tubular atrophy, while no Gd is detected in preserved renal tissue. Gd concentrations range widely, with some regions showing levels above 2000 ppm. The study also investigates the association between Gd and bio-metals such as calcium (Ca), zinc (Zn), copper (Cu), and iron (Fe). It finds no significant correlations between Gd and these metals at lower Gd concentrations, suggesting that a concentration threshold must be exceeded to observe interactions between Gd and bio-metals. This research provides valuable insights into Gd metabolism and its potential pathological effects in patients exposed to gadolinium-based contrast agents (GBCAs), particularly in those with renal impairments. The findings highlight the need for the further exploration of Gd retention and its long-term impact on kidney function.

Developments of stable and bright quantum dots offer a unique and feasible approach to image and visualize the cellular system. The article titled “Picomolar Detection of Lead Ions (Pb^2+^) by Functionally Modified Fluorescent Carbon Quantum Dots from Watermelon Juice and Their Imaging in Cancer Cells” describes a green, cost-effective approach to synthesize fluorescent carbon quantum dots (CQDs) using watermelon juice as the carbon source (Contribution 3). By functionalizing these CQDs with nitrogen-containing ligands, such as ethanolamine (EA) and ethylenediamine (ED), the fluorescence intensities were significantly enhanced. These functionalized CQDs (WMED CQDs) demonstrated a high sensitivity for detecting Pb^2+^ ions in polluted water, with a limit of detection as low as 190 pM. In addition to environmental applications, the study also highlights the ability of WMED CQDs to detect Pb^2+^ ions in live cancer cells (HeLa cells), emitting distinct fluorescence signals upon binding with Pb^2+^. The synthesized CQDs exhibit several desirable properties, including excitation-dependent emission, high photostability, and good solubility in water, making them ideal for applications in both biological and environmental samples for detecting heavy metal ions like Pb^2+^. This approach not only offers a sustainable method for preparing CQDs, but also presents promising applications for monitoring heavy metal contamination and imaging in biological systems.

A novel approach combining light sheet microscopy (LLS) with incoherent holography (IHLLS) has been developed to enhance spatiotemporal resolution while reducing photodamage in live cell imaging. This technique is applied to visualize AMPA receptor-mediated calcium transients in neuronal tissue. The IHLLS method employs both high and low spatial coherence, allowing for detailed imaging of subcellular structures and dynamic cellular processes. By reducing spatial coherence selectively, the technique minimizes phototoxicity and improves image quality, making it ideal for the long-term live imaging of delicate neuronal tissues. The study demonstrates the utility of IHLLS in providing high-resolution, real-time insights into cellular signaling within intact tissue (Contribution 4). This technique offers significant advancement in neuroscience, as it allows researchers to study neuronal signaling processes in great detail without the adverse effects of high-intensity light exposure, which is particularly beneficial for preserving live cell integrity during imaging. The IHLLS approach opens new pathways for investigating the spatiotemporal aspects of neuronal communication and other complex biological processes.

An innovative microscopy technique combining confocal autofluorescence and optoacoustic imaging has been developed for the early and label-free detection of pathogen infections in plant tissues. This hybrid approach (Contribution 5) has proven effective in identifying physiological changes in plant tissues infected by pathogens like *Xanthomonas campestris pv. campestris* well before visible symptoms emerge. In the study, optoacoustic signals were detected in broccoli leaves just 24 h after inoculation, far earlier than the typical appearance of visible symptoms. This method offers significant advantages over traditional plant disease detection techniques, particularly for quarantine pathogens such as *Xylella fastidiosa*. By detecting infections in their early stages, the technique allows for timely intervention, which can help prevent the spread of plant diseases. The combined use of autofluorescence and optoacoustic microscopy also provides a non-invasive, rapid detection approach that could be applied broadly to monitor plant health and manage agricultural pathogens more effectively. This research highlights the potential of hybrid imaging techniques to revolutionize plant pathology diagnostics, offering a powerful tool for both research and agricultural management.

The article “Unravelling the Mystery inside Cells by Using Single-Molecule Fluorescence Imaging” focuses on the significant advancements in live cell imaging, particularly at the single-molecule level (Contribution 6). It provides an in-depth review of how spatiotemporal nanoscale imaging allows researchers to investigate essential biological processes, such as protein–protein interactions, membrane dynamics, intracellular transport, gene expression, and organelle tracking. The article highlights the evolution of single-molecule tracking (SMT) through fluorescence microscopy, discussing its applications in studying cellular behavior and molecular dynamics with high precision. The review covers a range of topics, including improvements in labeling techniques, multi-channel detection, and time-lapse imaging, which have significantly advanced the field of single-molecule tracking. It also addresses the limitations of current techniques, such as phototoxicity and limited spatial and temporal resolution, and explores how advanced methods like super-resolution microscopy (SRM) and total internal reflection fluorescence microscopy (TIRFM) help to overcome these challenges. In addition to its contributions to basic biological research, SMT is also discussed in the context of medical diagnostics, offering the potential for the early detection of diseases by monitoring protein interactions and other cellular behaviors. The article concludes by discussing the future directions of SMT, focusing on the need for further technological improvements and broader applications in life sciences. The paper emphasizes that single-molecule imaging is crucial for deepening our understanding of complex cellular processes and offers promising avenues for both research and clinical applications.

A recent study has investigate the application of near-infrared fluorescence (NIRF) imaging in preclinical glioblastoma models to improve tumor detection and monitoring (Contribution 7). By utilizing three different fluorescent markers—IRDye 800CW conjugated with RGD, PEG, and 2-DG—the researchers explored each marker’s capacity to target distinct tumor characteristics such as αvβ3 integrin expression, enhanced permeability and retention (EPR) effects, and glucose metabolism. The most significant results came from IRDye 800CW RGD, which successfully visualized tumor sites with high specificity, as it binds to αvβ3 integrins, which are commonly overexpressed in glioblastoma. This allowed for a clear distinction between tumor tissue and healthy tissue, both in vivo and ex vivo. PEG and 2-DG markers were less effective, particularly in differentiating tumor tissue in deeper brain structures and peripheral regions of the tumor. The research highlights the potential of NIRF imaging, especially with RGD markers, as a non-invasive tool for tracking tumor progression and evaluating treatment efficacy. The deep tissue penetration and high sensitivity of NIRF imaging make it a promising technique for future cancer diagnostics and personalized therapy, particularly in aggressive cancers like glioblastoma. Further studies are recommended to optimize the timing and application of these markers for broader use in clinical settings.

Neuroactivity Toolkit is a toolbox designed for the quantitative analysis of neuronal network activity using miniscope fluorescence microscopy data from freely moving animals (Contribution 8). The tool offers a comprehensive set of statistical metrics to measure neuronal activation patterns, co-active neuron pair correlations, and network-wide properties, allowing researchers to explore how neuronal networks function in various conditions like learning, stress, and disease. The NeuroActivityToolkit simplifies the analysis of miniscope recordings by providing user-friendly interfaces for extracting data, calculating metrics like burst rate and spike duration, and visualizing neuronal network states. It also offers dimensionality reduction via PCA and includes shuffling techniques to validate the biological relevance of observed patterns by comparing with randomized data. The study emphasizes the potential of the toolkit for analyzing neuronal activity in the context of neurodegenerative disorders and behavioral studies. The open-source nature of the toolbox and the accompanying detailed tutorial make it accessible and practical for a wide range of experimental setups, improving researchers’ ability to extract meaningful insights from large neuronal datasets.
